# Hair microscopy: an easy adjunct to diagnosis of systemic diseases in children

**DOI:** 10.1186/s42649-021-00067-6

**Published:** 2021-11-29

**Authors:** Dharmagat Bhattarai, Aaqib Zaffar Banday, Rohit Sadanand, Kanika Arora, Gurjit Kaur, Satish Sharma, Amit Rawat

**Affiliations:** Allergy Immunology Unit, Department of Pediatrics, Advanced Pediatrics Centre, Post Graduate Institute of Medical Education and Research (PGIMER), Chandigarh, 160012 India

**Keywords:** Diagnosis, Disease, Hair, Microscopy, Primary health care

## Abstract

Hair, having distinct stages of growth, is a dynamic component of the integumentary system. Nonetheless, derangement in its structure and growth pattern often provides vital clues for the diagnosis of systemic diseases. Assessment of the hair structure by various microscopy techniques is, hence, a valuable tool for the diagnosis of several systemic and cutaneous disorders. Systemic illnesses like Comel-Netherton syndrome, Griscelli syndrome, Chediak Higashi syndrome, and Menkes disease display pathognomonic findings on hair microscopy which, consequently, provide crucial evidence for disease diagnosis. With minimal training, light microscopy of the hair can easily be performed even by clinicians and other health care providers which can, thus, serve as a useful tool for disease diagnosis at the patient’s bedside. This is especially true for resource-constrained settings where access and availability of advanced investigations (like molecular diagnostics) is a major constraint. Despite its immense clinical utility and non-invasive nature, hair microscopy seems to be an underutilized diagnostic modality. Lack of awareness regarding the important findings on hair microscopy may be one of the crucial reasons for its underutilization. Herein, we, therefore, present a comprehensive overview of the available methods for hair microscopy and the pertinent findings that can be observed in various diseases.

## Key points

1. Hair microscopy is an underrated but important supporting tool in diagnosing various types of diseases.
2. Rapidly evolving technologically advanced microscopic examination methods are available for hair examination.
3. Many congenital and acquired diseases/syndromes result in peculiar changes in hair structure and growth.
4. Some hair features (e.g. in Griscelli syndrome, Menkes disease) are pathognomonic of the disease which can be discerned with simple hair examination.

## Introduction

The attainment of a definitive (e.g. molecular) diagnosis is often challenging in resource-poor settings due to technological and logistic constraints. Microscopic examination of the hair (also called hair microscopy) is an inexpensive tool that may aid in the diagnosis of systemic disorders as the appearance, structure, and/or growth of hair are modified in various congenital and acquired diseases. In some diseases, pathognomonic findings may be appreciated on hair microscopy (e.g. Chediak Higashi syndrome (CHS), Menkes disease); while in others, the findings may provide valuable supportive evidence (e.g. kwashiorkor, biotinidase deficiency) (Tümer and Møller 2010; Inamadar et al. 2011). Even in resource-poor settings, hair microscopy may be underutilized by pediatric healthcare providers in the diagnostic evaluation of patients with systemic diseases. Lack of awareness regarding the important findings on hair microscopy may be one of the crucial reasons for its underutilization. Herein, we provide a brief overview of important systemic diseases wherein hair microscopy provides valuable clues to diagnosis. Besides, we briefly discuss the methods applied for hair microscopy.

### Normal structure of human hair

The hair follicle is divided into outer and inner portions. The outermost portion adjoining with epidermis and the stem cell bulge is the outer root sheath. The inner root sheath comprises Henle’s layer, Huxley’s layer, and cuticle (Goodier and Hordinsky 2015). The inner sheath acts as a supporting foundation for fiber shape, cortex, and cuticle (Hashimoto 1988). The hair shaft comprises a peripheral 6-layered cuticle, cortical layer (medial and concentric to cuticle), and medulla. Cuticle through its cysteine and ε-amino-(γ-glutamyl) lysine content provides physical probity, uprightness, and solidarity to the shaft (Goodier and Hordinsky 2015). The cortex comprises keratin proteins, containing integrating disulfide bonds between cysteine moieties, and keratin-associated proteins which merge and organize into the mesh of mid-zone filaments. (Goodier and Hordinsky 2015). Tensile strength is ensured through these proteins and filaments. (Shimomura and Ito 2005). The most central concentric medullary layer is not universally present, lacks keratin strands, and is prone to genetic changes and inter-individual inconsistency. Generally, its size relates to the dimension of the hair fiber (Das-Chaudhuri 1976).

### Phases of hair growth

Hair grows in 3 distinct stages: growing phase (anagen), transition phase (catagen), and rest phase (telogen). Though the exact molecular mechanism to control these phases is not known, modulation of stem cells by growth factors and cytokines might contribute to these transitions. Hair follicles are also among the few immune-privileged sites of the body which escape immune surveillance. The density and characteristics of hair growth may be influenced by race, ethnicities, hormonal influence, genetic polymorphisms, and epigenetics (Ocampo-Garza et al. 2019; Juhász and Atanaskova Mesinkovska 2020).

## Techniques applied for the evaluation of hair shafts

### Dermoscopy

Dermatoscopy, also known as dermoscopy, finds its roots in the 1600s when the concept of skin surface microscopy was primarily used for the examination of nail fold capillaries. It was only in the early to mid-twentieth century that the technique was applied to other skin lesions by Johann Saphier and Leon Goldman (Berk-Krauss and Laird 2017). Dermatoscopy is now widely recognized as an invaluable non-invasive tool in the evaluation of the surface features of the skin (Nirmal 2017). It is especially helpful in the early identification of malignant cutaneous lesions which may not appear so to the naked eye. Application of dermatoscopy to scalp and hair is termed trichoscopy. Trichoscopy enables visualization of hair shafts and their characterization (Rudnicka et al. 2008) and may supersede light microscopy in examining hair of suspected patients (Rudnicka et al. 2013).

### Light microscopy

Samples can be collected for light microscopy by clipping the hair close to the scalp (for studying the shaft) or plucking of hair with rubber-tipped forceps (when the roots need to be studied) (Inamadar et al. 2011). A series of lenses are used to magnify the specimen 10 to 1000 times with a resolving power of 0.2 μm depending on the type and numerical aperture. Structural hair shaft defects, fractures, nodes, bands, narrowing, twists, and curls can be readily identified by light microscopy; however, more invasive sample collection and complex preparations are required in some conditions. Light microscopy of the hair shaft can easily be performed, even at the patient’s bedside, by mounting 5–10 mm of hair shafts on glass slides using dibutylphthalate polystyrene xylene (DPX). It has already proven to be very useful in documenting characteristic abnormalities that accompany various systemic disorders in children (Smith 2005; Shao and Newell 2014). However, non-specific changes such as weathering and splits in distal shafts, twisting, mild flattening, or grooving may also be noted.

### Polarized light microscopy

Polarized light microscopy (PLM) is based on the principle of differential refraction of polarized light which results from local anisotropy of the specimen’s refractive index, absorption coefficient, and orientation to the polarization of light (Oldenbourg 2013). Hence, polarized microscopes have additional components compared to a simple light microscope, especially 2 sets of polarizers. The first polarizer is placed between the source of light and the specimen to ensure that the light illuminating the specimen is polarized. The variable physical nature of the various macromolecules present in hair results in birefringence (Keefe et al. 2003). Phase difference, which is essential for birefringence, results from the anisotropy of the hair shafts hence generated. While passing through the hair shaft, the speed of light which is polarized parallel to one axis, hence, differs from the speed of light which is polarized to the orthogonal axis (Oldenbourg 2013). A second polarizer (called analyzer) precedes the objective of the microscope and, when turned perpendicular to the light source, only transmits the light polarized by the birefringent hair shaft giving it a bright appearance (Pomeranz et al. 2013; Koike-Tani et al. 2015). Any light that passes through without any change induced by the hair specimen is hence eliminated.

### Electron microscopy

Electron microscopy employs beams of electrons directed at a suitably prepared specimen. As compared to light, electron beams have a much shorter wavelength which results in a significantly higher resolution in electron microscopy (Bravman and Sinclair 1984; De Cássia Comis Wagner et al. 2007). Transmission electron microscopy (TEM) is invaluable in visualizing the ultrastructure of cells (Winey et al. 2014). In hair microscopy, it is mainly used for studying the hair follicle. However, the hair sample needs processing including resin embedment before visualization by TEM (De Cássia Comis Wagner et al. 2007). Scanning electron microscopy (SEM) is more suited for studying the hair shaft and allows high-resolution 3-dimensional reconstruction of the surface features at magnifications up to 20,000 times. It can also detect subtle changes induced by cosmetic treatment of hair such as bleaching, dyeing, and straightening (Dawber 1970; Kaliyadan et al. 2016). In contrast to other materials (which need to be fixated to be viewed using SEM), the hair shaft being a fairly rigid and dry structure does not need fixation (Dawber 1970). However, sample preparation requires vacuum exposure and metallization step for analysis by SEM and these steps can induce changes in the surface of hair (Poletti et al. 2003).

### Atomic force microscopy

Atomic force microscopy (AFM) detects the physical interaction of a minute tip that is mounted on a cantilever over the specimen of interest. The cantilever deflections produced by this interaction are processed to produce images with atomic resolution. AFM does not require any specific pretreatment. Hence, this technique can be used to study the hair shaft when effects from sample modification are suspected (Poletti et al. 2003). It can be used to study live cells and is operational under liquids also. AFM is also used to visualize sample surfaces (Rugar and Hansma 1990). Its application to hair shafts led to the recognition of new surface features of the cuticle, rough surface, and step discontinuities on scale surfaces that were not apparent on SEM (Swift and Smith 2006; Trache and Meininger 2008). AFM can also be used to determine the accurate thickness of the hair shaft which is not possible in electron microscopy (You and Yu 2006).

### Confocal scanning microscopy

Confocal microscopy allows imaging of a single spot in the sample on which the illumination and detection optics are focused. This is of great benefit as it reduces blur due to light from different depths within the sample and significantly increases the resolution of the images collected. Data is collected point by point by shifting the focus of the optics, which can then be used to reconstruct 3D models of the specimen. The advantages of this method over SEM are that it requires minimal sample preparation, allows observation of hair in their natural environment, and causes minimal damage to the specimen (Hadjur et al. 2006). It can be used in reflection and fluorescent modes to study the surface morphology and internal structure of hair respectively. Confocal scanning microscopy (CSM) can be used to study the surface of the cuticle, identify and quantify exogenous deposits, and obtain high-quality 3-D images of hair (Lagarde et al. 1994; You and Yu 2006; Hadjur et al. 2006). An example of the use of CSM would be the study of dyed hair which shows the dye to remain in the periphery of the hair shaft, primarily in the endocuticle (Formanek et al. 2006). It is often used to study the effects of cosmetic treatment.

## Systemic diseases and hair microscopy

### Comèl-Netherton syndrome

Comèl-Netherton syndrome (CNS) is an autosomal recessive inborn error of immunity (IEI) classified under the hyper immunoglobulin E (IgE) syndrome group of combined immunodeficiency disorders in the recent *International Union of Immunological Societies* classification (Tangye et al. 2020). Mutations in the *SPINK5* (serine protease inhibitor, Kazal type 5) gene cause CNS and, to date, approximately 200 patients have been reported world over (Sarri et al. 2017; Bellon et al. 2021). *SPINK5* gene encodes for LEKTI (lymphoepithelial Kazal-type-related inhibitor) protein which inhibits the kallikreins (serine proteases) in the skin. In simple terms, defects in LEKTI protein result in increased activity of kallikreins that lead to degradation of desmosomes in stratum corneum, and increased skin permeability (Guevara-Patiño and Plaza-Rojas 2020).

Clinical manifestations include eosinophilia, elevated IgE levels, predisposition to allergic disorders, and recurrent infections of skin, respiratory and gastrointestinal systems. The infecting microbes reported include *Staphylococcus aureus* (most common), *Pseudomonas aeruginosa*, and members of the Enterobacteriaceae family amongst others (Renner et al. 2009). Besides, ectodermal abnormalities are an integral part of this disorder manifesting as erythroderma, ichthyosis, and hair abnormalities. A recent article describes the loss of skin barrier function as the primary pathogenetic mechanism leading to infections and allergic diathesis in CNS, contrary to the previously held notion of a systemic immunological abnormality (Stuvel et al. 2020).

Visualization of the characteristic hair shaft abnormality in CNS, called “trichorrhexis invaginata” or “bamboo hair”, can be a useful adjunct to the diagnosis. As the name suggests, CNS results in folding or telescoping of a segment (resembling intussusception seen in bowel loops) of hair into another, which can occur at multiple points in the hair shaft resembling a bamboo stick. Breakage of these anomalous hairs near the invaginating portion leads to a hair shaft with bulbous broken hairs called “golf-tee” or “match-stick” hairs (in case of more prominent bulbosity) (Rudnicka et al. 2018). This abnormality is visualized through various techniques namely videodermoscopy (trichoscopy), light and polarized microscopy, high-magnification histopathology, and scanning electron micrography. In low magnification settings, the telescoping segment may just be visualized as a nodular region or not visualized at all. In these settings, polarized microscopy may have an increased sensitivity by displaying an inhomogenous “band-like” pattern generated due to the self-folding of the hair shaft (Utsumi et al. 2020). SEM provides a high resolution, high magnification 3-dimensional visualization of the hair shaft abnormalities. Additional non-pathognomonic abnormalities like trichoptilosis or trichoschisis, which represent longitudinal and transverse breaks in the hair shaft respectively, may also be better visualized by this technique (Solovan et al. 2015). Patients with CNS may also have trichorrhexis nodosa, pili torti, and other abnormalities that are not pathognomonic for the disease (Rudnicka et al. 2013; Utsumi et al. 2020). Microscopy of the eyebrow hair is described to be of the highest sensitivity as compared to scalp hair or eyelashes in picking up trichorrhexis invaginata and visualization of a single hair with this finding is diagnostic of CNS (Powell et al. 1999).

### Griscelli syndrome type 1

Griscelli syndrome type 1 (GS1), an autosomal recessive disorder, results from mutations in *MYO5A* gene on chromosome 15q21, which plays a role in melanosome transport and membrane trafficking processes (Abd Elmaksoud et al. 2020). Myosin 5a, which is the product of *MYO5A* gene, also plays has a role in the control of inositol 1,4,5-triphosphate-mediated signaling as well as mRNA transport within neurons and synaptic activity (Çağdaş et al. 2012).

Unlike the uncontrolled hemophagocytic lymphohistiocytosis (HLH) of type 2 and partial albinism of type 3, GS1 has prominent central nervous system involvement with severe developmental delay, hypotonia, seizures, and ophthalmological involvement such as diplopia, nystagmus, and retinal problems. Other findings on physical examination include silvery gray hair, eyebrows, and eyelashes, with large pigment clumps identifiable on hair shafts under a light microscope.

### Griscelli syndrome type 2

Griscelli syndrome type 2 (GS2) is a familial autosomal recessive HLH syndrome very similar to CHS in its clinical features and disease pathogenesis. It is caused by mutations in *RAB27A* gene (Tangye et al. 2020). The Ras-related protein Rab-27A regulates intracellular traffic more downstream to the LYST protein and is involved in more terminal steps of vesicle docking, priming, and fusion (Bowman et al. 2019).

Similar to CHS, clinical manifestations in GS2 are partial oculocutaneous albinism, increased predisposition to develop HLH and infections, neutropenia, thrombocytopenia, and hepatosplenomegaly. However, the absence of giant granules in neutrophils and other granulocytes (including precursors) in GS2 is a useful differentiating feature. Similarly, neurological involvement has been reported to be less common in GS2 (than seen in CHS) and attributed to be due to lymphocytic infiltration rather than the disease per se (Krzewski and Cullinane 2013; Bowman et al. 2019).

**Fig. 1:**
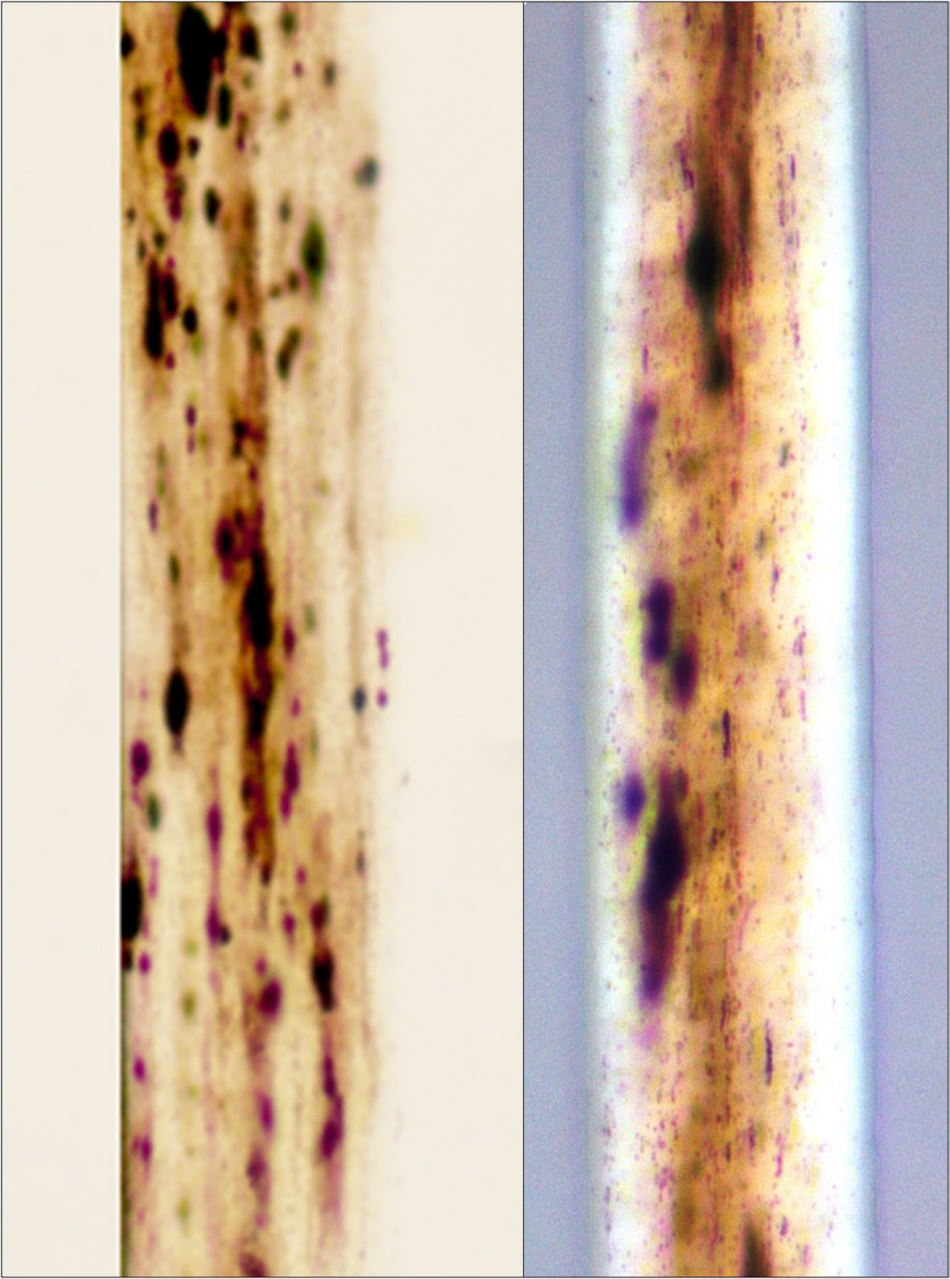
Hair microscopy findings in Griscelli syndrome type 2 visualized using a high power objective (40X); left panel: light microscopy showing large coarse clumps of melanin distributed in the cortex in an irregularly irregular pattern; right panel: polarized light microscopy showing uniformly white color of the hair shaft

Hair microscopy is also a very useful modality to differentiate GS2 from CHS. Light microscopy shows an irregularly irregular distribution of melanin granules in the hair shafts (Fig. 1) that are larger as compared to CHS and normal hair (Chandravathi et al. 2017). In addition, the granules are unevenly distributed across the thickness of the hair shaft (Fig. 1) and are localized more densely near the medulla. PLM shows a uniformly white color of hair shafts in GS2 (Fig. 1), probably because of minimal dispersion of light due to the irregular distribution and larger melanin granules in GS2 or nearly absent melanin granules in the cortex (Valente et al. 2006; Sandrock and Zieger 2010). TEM shows a similar irregular melanin granule distribution as is seen in light microscopy (Meeths et al. 2010). However, SEM demonstrates a normal cuticular pattern with occasional nodular keratin outpouchings (Celik et al. 2007). Recently, hematopoietic stem cell transplant is shown to also improve the hair pigmentation pattern in GS2 probably as a result of inadvertent specific donor stem cell engraftment near the hair follicles or non-specific drug-related side-effects or suppression of cytokine production which have an anti-melanogenic action (Yamazaki-Nakashimada et al. 2021).

### Chediak Higashi syndrome

CHS is an autosomal recessive primary immune dysregulatory disorder caused by mutations in the *LYST* (lysosomal trafficking regulator) gene (Tangye et al. 2020). This gene encodes for a large molecular weight protein that regulates intracellular vesicular transport, including lysosomes (Kaplan et al. 2008). The basic pathogenetic mechanism is the inability to reach specific targets by intracellular vesicles containing important molecules, like melanin in melanosomes, perforin, and granzyme in cytolytic vesicles, and serotonin adenosine nucleotides in platelet δ-granules (Kaplan et al. 2008; Nurden and Nurden 2011; Çağdaş et al. 2012). In addition, Lyst protein also participates in Toll-like receptor signaling inside innate immune cells (Westphal et al. 2017).

The principal clinical manifestations of CHS include partial oculocutaneous albinism, bleeding diathesis, giant granules in neutrophils and other granulocytes (including bone marrow precursors) with neutropenia, and an increased predisposition to infections and recurrent hemophagocytic lymphohistiocytosis. These patients may also have visual and other neurological disturbances (Kaplan et al. 2008).

**Fig. 2:**
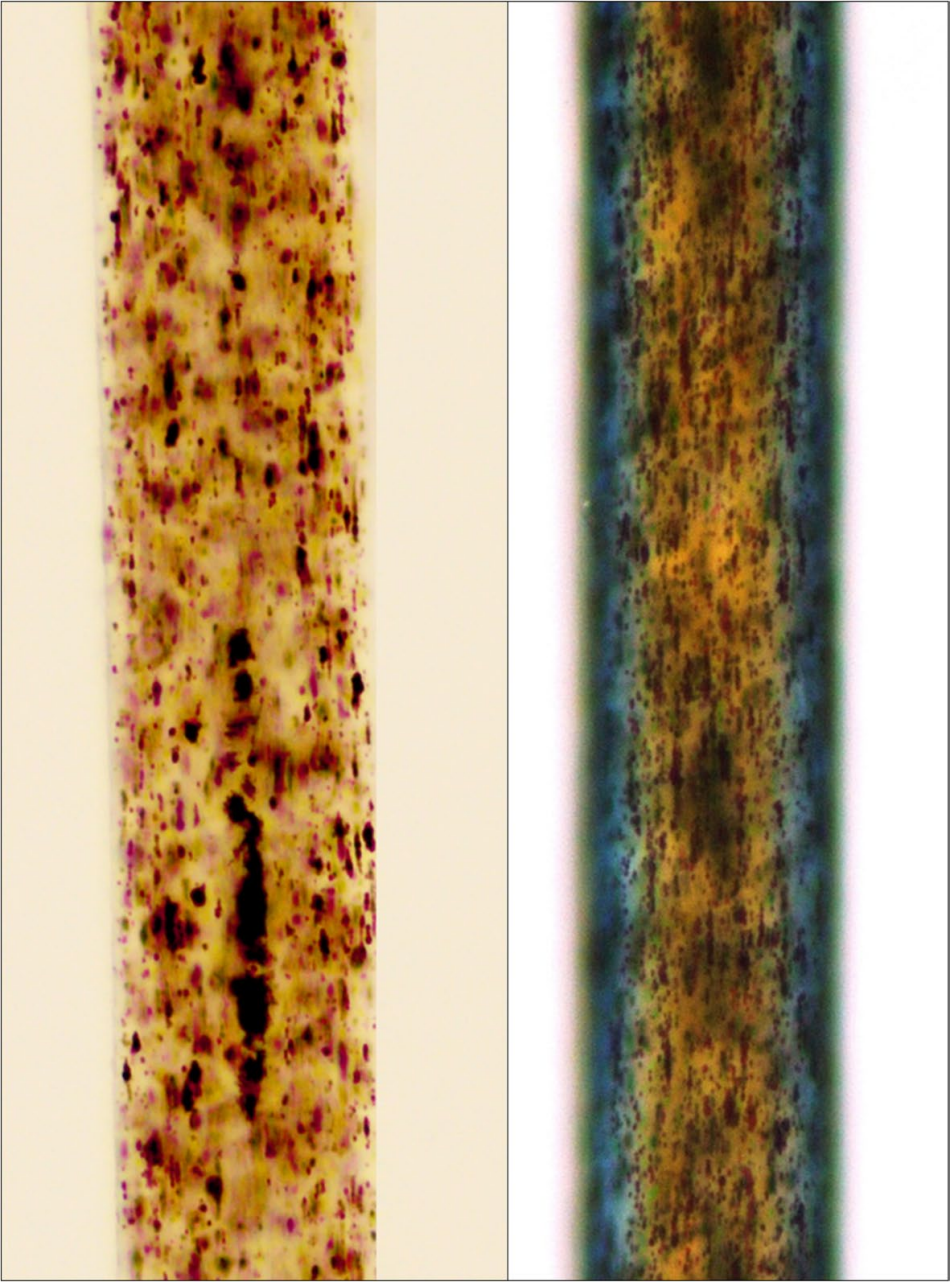
Hair microscopy findings in Chediak Higashi syndrome visualized using a high power objective (40X); left panel: light microscopy showing small fine clumps of melanin distributed in the cortex in a regularly irregular pattern; right panel: polarized light microscopy showing variegated colors of the hair shaft

Hair microscopy is valuable in the diagnosis of CHS as it helps to visualize abnormal pigmentation resulting from aberrant transport of melanin (contained in melanosomes) to the hair follicles. Light microscopy shows regularly irregular deposition of melanin granules (Fig. 2) which are larger than normal hair (Valente et al. 2006; Sandrock and Zieger 2010). PLM shows hair shafts with variegated colors (Fig. 2) resulting from the dispersion of light (Valente et al. 2006). Besides, granules in CHS not only have abnormal distribution in CHS but also have an abnormal shape (large and round, in contrast to normal ovoid shape) when visualized using TEM (de Almeida et al. 2018). Specific findings on trichoscopy or videodermoscopy, which can be performed without the need for removing hair, have not been reported to date in patients with CHS. This may be probably because of two reasons – lower resolution and magnification (up to × 1000 with high-end equipment) with video-dermoscopy as compared to light microscopy (Lacarrubba et al. 2015) or the ability to visualize the characteristic abnormalities with few hairs only, unlike CNS (Rudnicka et al. 2018).

### Trichothiodystrophy (TTD)

Price et al. coined the term trichothiodystrophy (TTD) (Greek: tricho-, thio-, dys-, and trophe- meaning disordered sulfur nourishment of hair) (Price 1980) for a group of maladies typified by sulfur-deficient fragile hair, intellectual disability, growth disturbances, ichthyosis, recurrent infections, reduced fertility, nail abnormalities, and features of premature aging. TTD is inherited in an autosomal recessive pattern. Three genes namely *XPB, XPD,* and *p8/TTDA* are found to be responsible for the photosensitive form of TTD (Stefanini et al. 2010). Some patients may also have hearing loss, dental caries, osteoporosis, cataracts, and severe infections leading to early death (Bergmann and Egly 2001). Distinct diagnostic criteria include hair shafts depicting an uneven contour and trichoschisis (fine vertical ridges), damaged cuticle and the end, flattened shaft, and trichorrhexis nodosa (Cheng et al. 2009). PLM shows a “tiger-tail” banding with alternating bands in addition to structural abnormalities. Amino acid analysis of hair shaft shows decreased sulfur and cysteine content (Price 1980).

Transilluminating trichoscopy under a polarized handheld dermoscope may be a useful alternative to PLM. The diagnosis of TTD should be made based on examination of all hair showing tiger tail pattern rather than few hairs. In a systemic review of 112 published cases, these distinct features on hair were present in all patients (Faghri et al. 2008). Brittle hair and abnormally damaged shaft were the most repeatedly observed finding (> 95%). Tiger tail banding, sparse hair, and reduced sulfur/cystine were illustrated in 73%, 48%, and 71% of patients, respectively. XPD mutations, decreased sulfur content, and tiger tail banding has also been described with the xeroderma pigmentosa (XP)/TTD complex in absence of frangible hair (Broughton 2001). Inflammatory and infectious causes were associated with hair loss in 8 patients of their cohort; (Chen et al. 1994; Broughton 2001; Faghri et al. 2008). Five patients were also reported to have long or normal length hair including 1 XP/TTD patient. Photosensitivity–ichthyosis–brittle hair–intellectual impairment–decrease fertility–short stature (PIBIDS) may also have TTD which is diagnosed on the basis of clinical examination and hair microscopy (Belloni Fortina et al. 2001).

### Malnutrition

Protein-energy malnutrition syndromes like kwashiorkor and marasmus may lead to brittle, curl-less, and sparse hair with hypochromotrichia (Bradfield 1973, 1981). In kwashiorkor, hairs are few, dry, and brittle. Flag sign, which represents alternate bands of lustreless and dark hairs, is typically seen. Since marasmus is a chronic adaptation to malnourished stress, hair follicles and roots go to the resting phase. There will be reduced hair tissue production in these children due to reduced protein synthesis. Shaft diameter and color of hair will also be reduced. The melanin content of hair is significantly reduced in malnourished children (McKenzie et al. 2007). In addition to changes in the hair follicle, malnutrition also leads to hair root changes. Generally, there is a reduction of pigment in the anagen phase leading to dysplasia, constrictions, roughness, or depigmentation. Kwashiorkor can also lead to broken hair roots. Significant bulb atrophy is seen in these patients.

In addition to malnutrition, specific macro- or micronutrient deficiencies may also lead to peculiar changes in the hair. Lack of ascorbic acid leads to curling, plugged follicles, corkscrew hair, and hyperkeratotic and hemorrhagic hair (Finner 2013). Biotin deficiency can cause structural changes like trichorrhexis nodosa. Cyanocobalamin deficiency may turn healthy hair lustreless and gray. Zinc deficiency is a well-known reason for brittleness, depigmented hair, and telogen effluvium (Guo and Katta 2017). Severe pellagra patients are noticed to suffer from diffuse hair loss in the progressive phase. Minor manifestation of lack of fatty acids displays decreased pigmentation and numbers of eyebrow and scalp hair. Iron deficiency can sometimes be notorious to cause banded thin anagen hairs and hair loss. It may be due to the contribution of iron for ribonuclease reductase required for dividing hair cells. Copper deficiency results in pili torti and hypopigmented hair (Finner 2013).

Lastly, in children with malnutrition, bulb diameter has been shown to correlate with the patient’s muscle bulk and anthropometric parameters (Johnson et al. 1976). Hair mineral analyses have also been proposed as a clinical guide for treating malnutrition (Han et al. 2016).

### Menkes disease

**Fig. 3:**
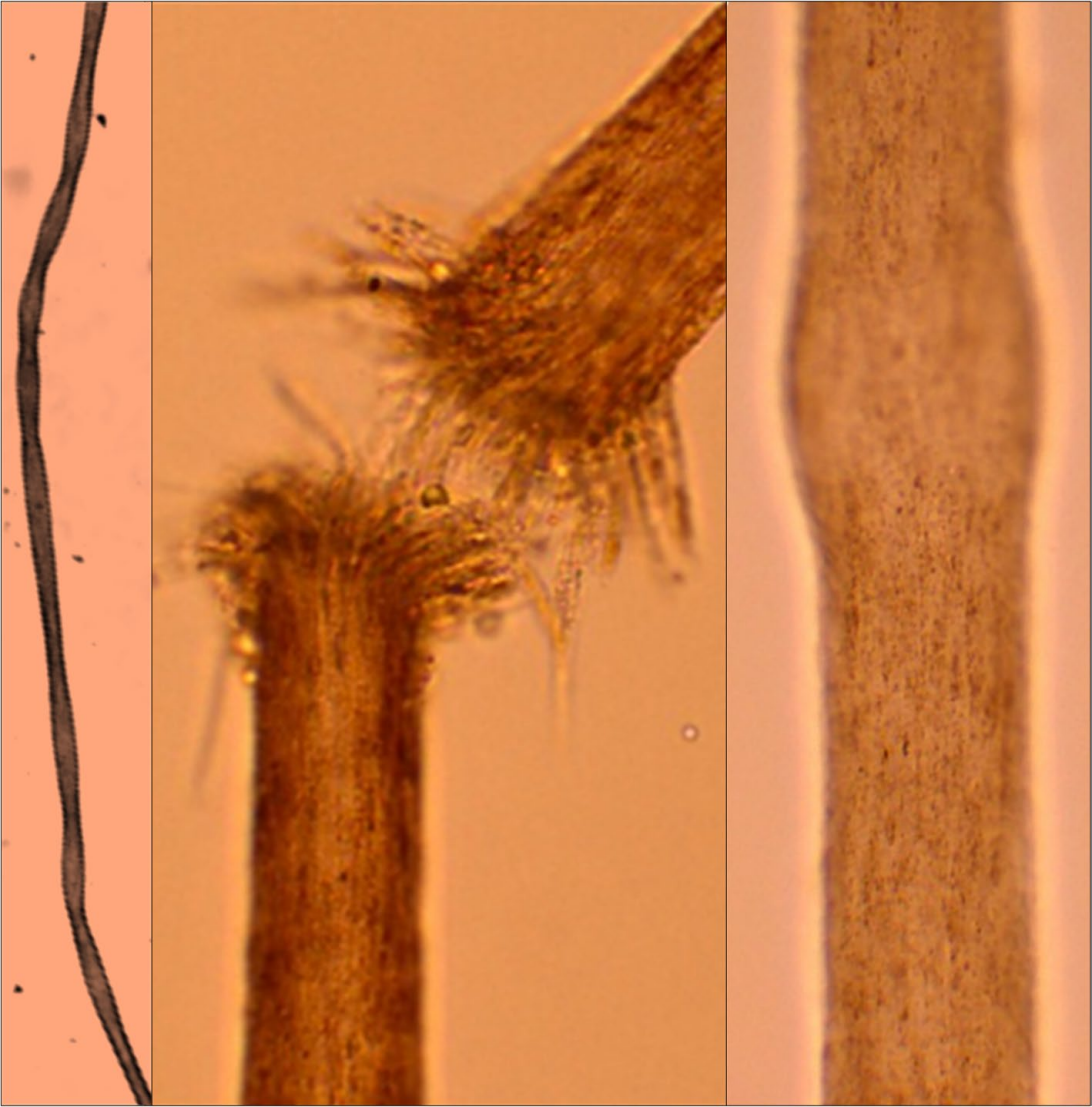
Light microscopy findings of hair in Menkes disease; left panel: low power view showing twisting of the hair shaft on its longitudinal axis called ‘pili torti’; middle and right panels: high-power views showing trichorrhexis nodosa

Menkes kinky hair disease is a multisystemic illness related to copper metabolism. Though the copper intake and uptake are normal in them, transport of copper to tissue is defective. Brain and hair changes are characteristically striking while evaluating patients with Menkes disease. Though scalp hair might look normal at birth, hair becomes characteristically kinky, stubby, depigmented, brittle, and mottled from mid-infancy or later (Choudhary et al. 2012). The kinky, tangled, stubby hypopigmented lustreless hair gives the feel of steel wool, hence the name kinky or steel hair disease (Tümer and Møller 2010). These patients may have generalized hair loss in course of time. Pili torti, a fully distorted hair on its axis is one of the common features found in hair microscopy (Fig. 3). Variable diameters of the hair shaft (monilethrix), fragmentation, proximally broken hairs (trichoclasis), chopped shaft, and fractured hair at intervals (trichorrhexis nodosa) are other typical findings (Fig. 3). Some of the hair follicles are completely within the dermis where short, fragile fragments of broken hair can be detected (Prasad and Ojha 2016).

### Monilethrix

Monilethrix is a rare hair shaft fragility genodermatosis (Sinclair 2016) resulting in patchy dystrophic alopecia. Dominantly inherited keratin mutations (with complete penetrance and variable expression) and recessively inherited desmoglein 4 mutations (resulting in more severe phenotype) have been identified in patients with monilethrix (Zlotogorski et al. 2006).

Affected individuals have normal hair at birth but develop fracture-prone brittle hair within few months of life. The severity of illness corresponds to the pattern of involvement, with severe disease involving even the eyebrows, eyelashes, and secondary sexual hair whereas the milder forms involving the scalp hair only. Trichoscopy reveals a pattern of regular variation in diameter of the hair shaft resulting in elliptical dilations (also called nodes), and constrictions (also called internodes), giving it a rosary beads-like appearance. Beaded hair-shafts lead to fragility at the internodal regions where medulla seems to be absent. Nodes occur at every 0.7–1 mm (Oliveira and Araripe 2015). There may be spontaneous improvement with puberty and pregnancy. In addition to hair abnormalities, follicular hyperkeratosis is a commonly observed finding.

Although there is no cure for this genetic condition, clinical response to low-dose minoxidil has been reported (Choudhary et al. 2012; Prasad and Ojha 2016). Measures to minimize hair trauma from cosmetic alterations are also important.

### Biotinidase deficiency

Biotinidase is an enzyme encoded by the *BTD* gene and is responsible for the re-usage of biotin from degraded carboxylases and dietary sources. Biotinidase deficiency is an autosomal recessive disorder caused due to mutations in the *BTD* gene, resulting in biotin dependency (Wolf et al. 1983). Biotin deficiency can also occur from prolonged consumption of raw eggs containing avidin and in patients receiving biotin-deficient parenteral alimentation and can have similar manifestations as biotinidase deficiency.

Biotinidase deficiency manifests with neurological and dermatological manifestations including seizures, developmental delay, hypotonia, alopecia, and dermatitis. It may manifest in the early weeks of life. Newborn screening can identify patients with this disorder before symptom onset, allowing supplementation of biotin and prevention of symptoms. Although the dermatological symptoms can be completely reversed by administering therapeutic doses of biotin, it is not so in the case of established neurological manifestations (Venkataraman et al. 2013). It is therefore important to look for metabolic causes in a child with neurological symptoms who also has hair abnormalities.

Early-onset alopecia is common in children with biotinidase deficiency and may manifest before the neurological symptoms. The hair microscopy finding seen is trichorrhexis nodosa, resulting from disruption of cuticular cells and node-like formation from the cortical cells (Coulter et al. 2008). The hair shafts have a “broomstick” deformity at their transverse fracture sites and show extreme fragility. Trichorrhexis nodosa is a non-specific finding often secondary to trauma induced by cosmetic alterations of the hair, but may also be congenital as seen in ectodermal dysplasias and other metabolic diseases such as argininosuccinic aciduria, citrullinemia, trichothiodystrophy, and Menkes syndrome (Burkhart and Burkhart 2007).

### Uncombable hair syndrome

Uncombable hair syndrome (UHS, also called pili trianguli et canaliculi), though first described in medical literature 1973, also appears to be talked about messy and uncombable hair of a German folk story character, Struwel Peter. It refers to an autosomal dominant syndrome, typified by straw-colored tardy growing scalp hair that is difficult to comb flat. The physical characteristics, including fiber thickness, tensile strength, and elasticity, as well as biochemical characteristics of individual strands, including amino-acid, sulfur, and copper levels are normal. The “uncombability” is thought to result from misshapen dermal papillae which harden, producing a rigid and abnormally shaped tube. However, hair root keratinization defects may also be contributory (Boccaletti et al. 2007).

The presentation is generally in early infancy due to dry, shiny, blonde hair that is difficult to comb. Ichthyosis, eczema, alopecia areata, and skeletal, dental and retinal abnormalities have also been reported in UHS (Calderon et al. 2009). In contrast to several other hair shaft disorders, light microscopic and dermoscopy findings are misleading as the hair appears normal. However, SEM reveals their bean or tripod-shaped cross-section and narrow tube-like depressions with an intact cuticle. This shape is thought to result from the greater bending rigidity of hair as compared to hair with a circular cross-section. Oral biotin supplementation has been reported to improve hair strength, growth rate, and combability without altering their triangular shape. Biotin is thought to alter the matrix proteins of the hair rather than keratin to yield favorable results in this condition (Boccaletti et al. 2007).

## Conclusion

Hair microscopy stands as a touchstone companion in the diagnostic journey of a wide array of local and systemic diseases which affect the normal growth and structure of the hair. Various technologically advanced methods for microscopic examination of the hair are available which are evolving rapidly with time. Nonetheless, light microscopy remains a useful, inexpensive, easily available technique to demonstrate peculiar changes in hair structure, many of which are notable for their diagnostic or pathognomonic importance. Besides, with minimal training, light microscopy of the hair can easily be performed even by clinicians which can, thus, serve as a useful tool for disease diagnosis at the patient’s bedside. This is especially true for resource-constrained settings where access and availability of advanced laboratory investigations (like molecular diagnostics) is a major constraint. Thus, hair microscopy, which may be underutilized by clinicians, should be considered in the diagnostic evaluation of various childhood disorders.

## Data Availability

All relevant data have been included in the manuscript itself.

## Abbreviations

AFM: Atomic force microscopy
CHS: Chediak Higashi syndrome
CNS: Comèl-Netherton syndrome
CSM: Confocal scanning microscopy
DPX: Dibutylphthalate polystyrene xylene
IgE: Immunoglobulin E
GS1: Griscelli syndrome type 1
GS2: Griscelli syndrome type 2
HLH: Hemophagocytic lymphohistiocytosis
PLM: Polarized light microscopy
SEM: Scanning electron microscopy
TEM: Transmission electron microscopy
TTD: Trichothiodystrophy
UHS: Uncombable hair syndrome
